# The HLA-G Immune Checkpoint Plays a Pivotal Role in the Regulation of Immune Response in Autoimmune Diseases

**DOI:** 10.3390/ijms222413348

**Published:** 2021-12-12

**Authors:** Monika Zaborek-Łyczba, Jakub Łyczba, Paulina Mertowska, Sebastian Mertowski, Anna Hymos, Martyna Podgajna, Paulina Niedźwiedzka-Rystwej, Ewelina Grywalska

**Affiliations:** 1 Department of Experimental Immunology, Medical University of Lublin, 4a Chodzki Street, 20-093 Lublin, Poland; monika.zaborekk@gmail.com (M.Z.-Ł.); jakublyczba7@gmail.com (J.Ł.); paulina.lipa56@gmail.com (P.M.); mertowskisebastian@gmail.com (S.M.); annahymos@gmail.com (A.H.); marpodgajna@gmail.com (M.P.); 2 Institute of Biology, University of Szczecin, 3c Felczaka Street, 71-412 Szczecin, Poland

**Keywords:** autoimmune diseases, human G-leukocyte antigen (HLA-G), major histocompatibility complex (MHC)

## Abstract

The human G-leukocyte antigen (HLA-G) molecule is a non-classical major histocompatibility complex (MHC) class I molecule. The pertinence of HLA-G has been investigated in numerous studies which have sought to elucidate the relevance of HLA-G in pathologic conditions, such as autoimmune diseases, cancers, and hematologic malignancies. One of the main goals of the current research on HLA-G is to use this molecule in clinical practice, either in diagnostics or as a therapeutic target. Since HLA-G antigens are currently considered as immunomodulatory molecules that are involved in reducing inflammatory and immune responses, in this review, we decided to focus on this group of antigens as potential determinants of progression in autoimmune diseases. This article highlights what we consider as recent pivotal findings on the immunomodulatory function of HLA-G, not only to establish the role of HLA-G in the human body, but also to explain how these proteins mediate the immune response.

## 1. Introduction

The human G-leukocyte antigen (HLA-G) molecule belongs to the class I histocompatibility antigens. Molecularly, it is a heterodimeric protein complex consisting of a heavy chain (containing eight exons) and a light chain in the form of beta-2 microglobulins [1,2]. The HLA-G gene is responsible for the synthesis of this molecule, which is located at the major histocompatibility complex (MHC) locus on chromosome 6. The HLA-G protein has special features that render it one of the non-classical HLA-I antigens. This applies to features such as: reduction in allelic polymorphism, reduction in tissue distribution, and the expression of seven isoforms represented by four membrane-bound proteins (G1, G2, G3 and G4) and the three soluble proteins (G5, G6 and G7). Both membrane-bound and soluble forms exert a number of immunomodulatory effects on the human body. These include the inhibition of the allogenic proliferation of CD4+ T cells, natural cytotoxicity of CD4+ (NK) cells and CD8+ T cells maturation. In addition, soluble HLA-G molecules are capable of inducing apoptosis in antigen-specific CD8+ T cells as well as in dendritic cells (DCs) and are involved in the activation of B cells [3,4,5,6,7,8,9]. It is also involved in the immune response and tolerance development during pregnancy. Under physiological conditions, HLA-G is expressed at high levels in the placenta, on trophoblasts, in the cornea, thymus, and pancreatic β islets, but at low levels in most mature cells. Scientific research shows that HLA-G expression can be induced under pathological conditions such as: neoplastic transformation, viral infections, inflammation, promoting transplant tolerance, and autoimmune diseases [10,11]. In the case of the latter, it is a deregulation of the immune system, which results in impaired tolerance to the autoantigen. It is estimated that many factors, such as genetic, environmental or hormonal ones, may directly or indirectly influence the development of autoimmune processes. Disturbances in the functioning of both innate and acquired immune response, which may be involved in the progression in this group of diseases, also play an important role [12]. However, the exact etiology and pathogenesis of autoimmune diseases is not fully understood. That is why it is so important to look for new molecules that can help explain how this kind of disorders occurs [10,11]. Because HLA-G antigens are currently considered immunomodulatory molecules that are involved in reducing inflammatory and immune responses in the human body, in this review, we decided to focus on this group of antigens as potential determinants of the progression of autoimmune diseases. Our goal is not only to estimate the role of HLA-G in the human body and to explain how these proteins mediate the immune response, but also to establish their participation in many autoimmune diseases such as: celiac disease, rheumatoid diseases, systemic sclerosis, systemic lupus erythematosus, asthma, or allergic rhinitis.

## 2. Molecular Characterization of the HLA-G Antigen

**Figure 1:**
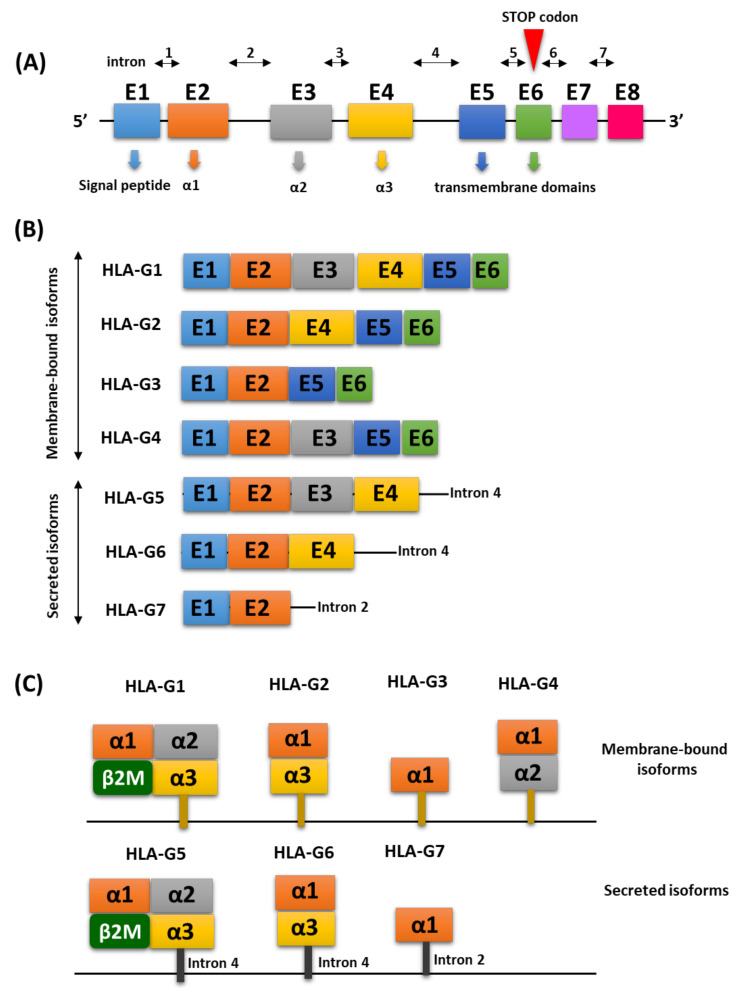
HLA-G genetic organization based on [21,22]: (**A**) genetic organization of the region responsible for HLA-G coding; (**B**) types of HLA-G isoforms with a particular emphasis on membrane-associated and secreted isoforms; and (**C**) spatial arrangement of the domains of individual HLA-G isoforms.

The human HLA-G protein is a heterodimer of 273 amino acids with a total molecular weight of 31.55 kDa. Its structure includes an Ig-like domain, which has 79 amino acids and is located between 184 and 262 amino acids [13]. The *HLA-G* gene located on chromosome 6p21 is responsible for coding this antigen. The structure of the HLA-G genes is homologous to other HLA class I (Ia) genes and consists of seven introns and eight exons coding the heavy chain of the molecule. Exon 1 is responsible for encoding the signal peptide, while exons 2, 3, and 4 are involved in encoding the extracellular domains: α1, α2, and α3. Exons 5 and 6 encode the heavy chain cytoplasm transmembrane domains [14,15]. Compared to classical class I molecules, HLA-G has a short cytoplasmic domain due to the presence of a premature stop codon in exon 6. Therefore, exon 7 is always absent in mature mRNA, while exon 8 is the three prime untranslated region (3′UTR) and is never subjected to translation (Figure 1A). The process of the alternate splicing of the mRNA transcription product provides seven isoforms of the HLA-G protein. HLA-G1, -G2, -G3 and -G4 are associated with the cell membrane, while the others (HLA-G5, -G6, -G7) belong to the soluble isoforms (Figure 1B). HLA-G1 is an isoform with the complete and typical structure of class I HLA related to β2-microglobulin containing exons 1–6 (Figure 1C). mRNA belonging to HLA-G2 lacks the α2 domain encoded by exon 3, while the HLA-G3 isoform lacks α2 domains and α3 encoded by exons 3 and 4, respectively, and HLA-G4 lacks the α3 domain encoded by exon 4 [16,17]. Soluble HLA-G5 and -G6 isoforms have been determined to contain the same domains as the HLA-G1 and HLA-G2 isoforms. HLA- G5 and -G6 are generated by intron 4 retaining transcripts that block the translation of the transmembrane domain (exon 5). Intron 4 is translated until the stop codon is read in its 5′ region, giving the HLA-G5 and HLA-G6 isoforms a 21 amino acid tail that contributes to their solubility. The HLA-G7 isoform only has the α1 domain fused to the two-intron two-encoded amino acids that are retained in the corresponding transcript. According to the literature, there are two molecules which are considered the most explored, important and functional HLA-G isoforms, namely HLA-G5 and HLA-G1. However, while HLA-G5 molecules are actively secreted as soluble isoforms, HLA-G1 proteins can be released from the cell surface during translation by matrix metalloproteinase 2 (MMP-2), resulting in a reduced soluble form of HLA-G (sHLA-G1) [18,19]. This antigen either exists as a monomeric protein (β2m bound), as dimers (linked by disulfide bonds between two cysteine residues at positions 42 and 147 of the heavy chain) or as multimers. Due to dimerization, the spatial structure of HLA-G transforms and binding sites on the α3 domain become more accessible to receptors and dissociate more slowly compared to monomers [20].

### Regulation of HLA-G Expression

**Figure 2:**
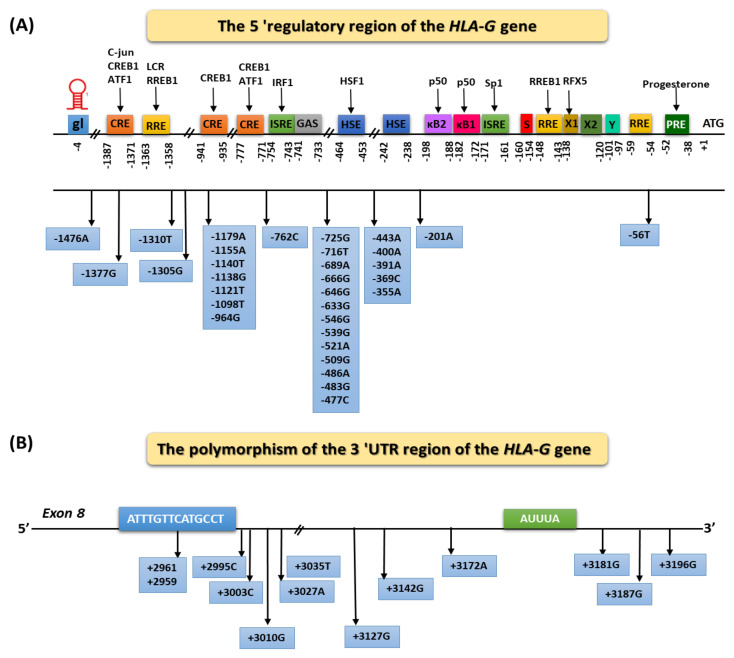
Regulation of HLA-G gene expression considering the genetic polymorphism of the SNP type based on [17,29,31,32,33,34,35,36]. (**A**) The 5′ regulatory region of the *HLA-G* gene; and (**B**) the polymorphism of the 3′UTR region of the *HLA-G* gene. Abbreviations: ATF1: cyclic AMP-dependent transcription factor ATF-1; C-jun: transcription factor encoded by the JUN gene; CRE: cAMP response element; CREB1: CAMP responsive element binding protein 1; GAS: interferon-gamma activated site; gL: LINE-1 retrotransposon at the HLA-G promoter; HRE: hypoxia response element; HSE: heat shock element; HSF1: heat shock factor 1; IRF1: interferon regulatory factor 1; ISRE: interferon-stimulated response element; LCR: locus control region (candidate); p50: nuclear factor NF-κ-B p105 subunit; PRE: progesterone response element; RFX5: DNA-binding protein RFX5 (RFX family); RRE: Ras response element; RREB1: Ras responsive element binding protein 1; Sp1: transcription factor Sp1 (also known as specificity protein 1).

HLA-G expression is regulated in both transcription and post-transcription processing. The exogenous factors increasing the expression of HLA-G include stress, fasting, hypoxia, as well as hormones and cytokines, e.g., progesterone, interleukin 10 (IL-10), granulocyte-macrophage colony stimulating factor (GM-CSF), interferons (IFNs), TNF-α, and transforming growth factor b (TGF-β). In addition, it is believed that epigenetic changes such as DNA demethylation and the inhibition of histone acetylation can lead to repressed HLA-G gene activation. Genetic conditions can influence individual differences in the expression of the HLA-G molecule [21,22,23]. It is also believed that the presence of certain polymorphic variants in the structure of the HLA-G gene may affect the level of its transcription. Genes encoding HLA-G show much less polymorphism than classical HLA molecules genes. To date, 72 single nucleotide polymorphisms (SNPs) located between exon 1 and intron 6 and 44 encoding alleles within HLA-G have been described. Thirty-three SNPs were identified in the heavy chain coding region, but only 13 of them changed the encoded amino acid: four in the α1 domain, six in the α2 domain, and three in the α3 domain [17,24]. Despite the low variability of HLA-G, changes in amino acids may affect the individual isoforms’ formation, their expression, and biological properties, such as the ability to bind peptides or to modulate the immune response. HLA-G production is controlled by several polymorphisms, both in the promoter and in the 3′ untranslated region (3′ UTR), modifying the affinity of the target genes for transcriptional or post-transcriptional factors, respectively [25,26]. The presence of certain sequences as well as polymorphic variants in the 5′ upregulating region of HLA-G (URR) may be of great importance in the regulation of HLA-G protein expression. The region between 1.1 and 1.4 kb from the transcription start site to which nuclear transcription factors bind is of particular importance. Twenty-nine SNPs were identified in the HLA-G promoter region. Functionally active SNPs have been located, among others, in the position −725 (G/C/T), −201 (A/G), and −964 (G/A) (Figure 2A) [17,24,27]. The guanine substitution at position −725 correlates with increased HLA-G expression. Literature data estimate that couples in which both partners possess the −725 G allele have a greater risk of recurrent spontaneous abortion than couples without this allele. On the other hand, the replacement of guanine with adenine at position −201 is associated with a change in the affinity of the nuclear transcription factor kB (NF-kB, kappa B) for enhancer A. It has also been shown that the presence of the GG genotype at position −964 correlates with a higher incidence of asthma among the children of mothers with this disease, while the AA genotype is associated with a higher incidence of asthma among children of healthy mothers [17,18].

In the expression regulation, HLA-G also has the 3′ UTR. Due to the “stop” codon present in the mature HLA-G mRNA at exon 6, the protein molecule lacks an exon 7. Due to this, exon 8 at the end remains untranslated and is a site containing many elements regulating transcription and translation. Adenine-rich and uracil-rich regions (AU-rich motifs) or adenine-rich regions (Poly-A) play an important role in these processes [28,29,30]. The presence of certain polymorphic variants in the 3′UTR region in the exon 8 may also affect the transcription, translation and expression of HLA-G at the protein level. Functionally active polymorphisms in this region include, but are not limited to, 14 base pair (bp) deletion/insertion polymorphisms (5′-ATTTGTTCATGCCT-3′) in exon 8, SNP at +3142 (C/G), SNP at +3172 (G/A) and SNP at +3187 (G/A) (Figure 2B). The presence of the sequence (5′-ATTTGTTCATGCCT-3′) in exon 8 is associated with decreased HLA-G protein expression, but at the same time with the greater mRNA stability for HLA-G lysoforms for both associated with the cell membrane and soluble forms [31,32,33,34].

At the HLA-G gene locus in the 3′UTR region, a total of eight different haplotypes (being a group of genes that an organism inherited from one parent expressed as a set of SNPs located on a single chromatid that are inherited as a set of linked alleles) were identified. It is believed that the haplotypes UTR-2, UTR-5, UTR-7, and UTR-8 correlate with lower mRNA stability, while higher mRNA stability is associated with the presence of the UTR-1 haplotype (Table 1) [35].

The literature reports indicate that +14 bp allele carriers and +14 bp/+14 bp homozygotes show a significantly lower HLA-G expression than in individuals without this genotype. There is evidence of an association between the presence of the 14 bp sequence and an increased risk of complications during pregnancy, such as recurrent spontaneous abortion or pre-eclampsia. A higher frequency of the +14 bp allele was also found in patients with systemic lupus erythematosus (SLE), which may suggest a relationship between this polymorphic variant and a predisposition to develop SLE. In addition, the missing sequence (5′-ATTTGTTCATGCCT-3′) has clinical implications. It has been proven that a 14 bp deletion in exon 8 results in a higher expression of the HLA-G protein. A correlation was demonstrated between the presence of the −14 bp allele and a higher risk of developing juvenile idiopathic arthritis [34,36,37].

**Table 1:** Examples of haplotypes on HLA-G 3′UTR based on [38].

Haplotype	Type of Mutation	Localization of Haplotype HLA-G in 3′UTR						
		+3003	+3010	+3027	+3035	+3142	+3187	+3196
UTR-1	Deletion	T	G	C	C	C	G	C
UTR-2	Insertion	T	C	C	C	G	A	G
UTR-3	Deletion	T	C	C	C	G	A	C
UTR-4	Deletion	C	G	C	C	C	A	C
UTR-5	Insertion	T	C	C	T	G	A	C
UTR-6	Deletion	T	G	C	C	C	A	C
UTR-7	Insertion	T	C	A	T	G	A	C
UTR-8	Insertion	T	G	C	C	G	A	G

It has also been found that many microenvironmental factors can increase the expression of HLA-G, such as cell stress, malnutrition, hypoxia, and the presence of progesterone or the participation of cytokines (GM-CSF, IL-10, TNF, TGF-β, interferons) [1].

## 3. Contribution of the HLA-G Antigens to the Immune Response

### 3.1. Characterization of Receptors for the HLA-G Molecule

There is evidence in the literature suggesting that HLA-G plays an important role as a tolerance-inducing molecule in suppressing the immune response. The extracellular domains of the HLA-G molecule affect specific immune cells by binding to receptors on their surface. HLA-G binds mainly receptors, i.e., ILT2 (immunoglobulin (Ig) -like transcript 2)/CD85j, ILT4 (Ig-like transcript 4)/CD85d, KIR2D4L (killer inhibitory receptor)/CD158d), CD8 and in recent years also CD160 described as the HLA-G receptor (Table 2) [2,39,40].

A characteristic feature of ILT2 and ILT4 receptors is the presence of ITIMs (tyrosine-based immunoreceptor inhibition motif) in their structure, which have a consensus sequence: S/I/V/LXYXXI/V/L. The KIR2DL4 receptor, in addition to the ITIM domain, has a second ITAMs domain (tyrosine-based immunoreceptor activation motif), with the following consensus sequence: YXXI/LX_6–12_YXXI/L. For both motifs, X translates to any amino acid present in the consensus sequence. Additionally, there are phosphorylation motifs found in many receptors or adapter proteins. The difference between the two motifs is that phosphorylated ITAMs serve as docking sites for the tandem SH2 domains of the Syk family kinases, while phosphorylated ITIMs are responsible for the recruitment of tyrosine phosphatases. These differences also translate into their functioning in the cell. Literature data indicate that signaling through receptors with the ITAM domain usually results in cell activation, while the involvement of receptors with an ITIM domain has the opposite effect [41,42].

ILT2 is expressed by the B cells, some of the T cells, certain NK cells, and all the monocytes/dendritic cells; however, ILT4 is myeloid specific and is only expressed by the monocytes/dendritic cells. Regarding KIR2DL4, it was found that its expression is mainly restricted to a subset of CD56 NK cells, which are a minority of peripheral NK cells but the majority of uterine NK cells. Nevertheless, through these differentially expressed receptors, HLA-G can interact with B cells, T cells, NK cells, and antigen-presenting cells [43,44,45,46,47,48,49]. ILT2 and ILT4 are clearly inhibitory receptors while the division is not so well defined for KIR2DL4. Indeed, it appears that KIR2DL4 can send both inhibitory and activating signals by having a single tyrosine-based immunoreceptor motif in the cytoplasmic tail and positively charged arginine in the transmembrane region [44,46]. The difference between ILT and KIR2DL4 is that ILT2 and ILT4 bind to classical HLA molecules, while HLA-G is the only ligand of KIR2DL4. HLA-G, however, is the ligand with the highest affinity for ILT2 and ILT4, and ILT2 and ILT4 have an even greater affinity for HLA-G multimers. Finally, ILT2 and ILT4 differ in the level of recognized HLA-G structures: ILT2 is the receptor for beta2-microglobulin-bound HLA-G, while ILT4 also recognizes HLA-G free heavy chains. Due to the interaction with ILT2 HLA-G receptors, it inhibits the cytolysis, proliferation and transmembrane migration of NK cells as well as the cytotoxic activity and proliferation of T cells [45,46,47,48]. The HLA-G molecule inhibits the maturation of dendritic cells and the activity of antigen-presenting cells (APC) by binding to ILT4 receptors. The HLA-G-mediated inhibition of NK cells has been extensively studied since NK cells are the major subset of cells involved in fetal and maternal tolerance. In vitro studies have shown that the interaction of ILT2 with membrane-bound or soluble HLA-G5 generates negative signals, resulting in NK cell-mediated lysis inhibition. Conversely, the binding of soluble HLA-G to the intracellular KIR2DL4 receptor does not affect NK cytotoxicity but induces the production of proinflammatory and proangiogenic cytokines and chemokines [49,50,51,52]. The reverse effect exerted by binding to CD160 receptors leads to apoptosis of epithelial cells, thus inhibiting neoangiogenesis. Soluble HLA-G isoforms also increase the expression of the CD95 molecule and lead to the activation of the apoptosis of T lymphocytes and NK cells through the Fas/FasL dependency mechanism [52,53]. Nevertheless, Le Page et al. questioned the presence of a functional interaction between soluble HLA-G and KIR2DL4 on NK cells. Therefore, it remains controversial whether the HLA-G/KIR2DL4 interaction influences NK cell activity [54,55]. Fons and colleagues showed that the interaction between soluble HLA-G and CD160 anchored to glycosylphosphatidylinositol on endothelial cells leads to the apoptosis of the latter cells and the inhibition of angiogenesis. However, CD160 is expressed not only on endothelial cells but also on NK cells, NKT cells, and T cells [56,57].

### 3.2. The Role of HLA-G in Physiological Conditions

The HLA-G molecule exhibits immunosuppressive properties that significantly inhibit the stages of the immune response, both at the level of differentiation, proliferation, cytolysis, and the secretion of cytokines and immunoglobulins. This is possible through the interaction of HLA-G with all immune cells that contain ILT-2, ILT-4 and KIR2DL4 receptors on their surface (Table 3). Thus, HLA-G molecules are involved in many immune response processes such as antigen presentation to T lymphocytes, the inhibition of dendritic cell differentiation, the inhibition of neutrophil-mediated phagocytosis, and the process of developing the immune tolerance [58,59].

Behind the immune tolerance process, there are several complex mechanisms that aim to eliminate foreign antigens, preventing damage to the host’s tissues. According to the literature, we distinguish two types of immunological tolerance: central (occurring during the development of lymphocytes in primary lymphoid organs) and peripheral (occurring in peripheral tissues and lymph nodes). The process of immune regulation is crucial for the maintenance of peripheral tolerance in which regulatory T cells (Treg) are involved. They play an important role not only in homeostasis, but also in the development of infectious diseases, cancer, or autoimmune diseases [1,58,59]. Research conducted by Feger et al. showed the existence of new subsets of T cells that express the immunomodulatory molecule HLA-G on their surface, identifying them as separate subpopulations of Treg lymphocytes [60]. Classic Tregs have the phenotype of CD4+ CD25+ FoxP3+, while HLA-G+ Treg cells do not have a forkhead box P3 (FOXP3), CD39, and CD25. Literature data indicate that the thymus is the reservoir of human HLA-G+ Treg cells, and their peripheral blood concentration in healthy people ranges from 0.1 to 8.3%. An important feature of these cells, compared to classic Tregs, is their low proliferation capacity. HLA-G+ Treg cells exert their suppressive functions by secreting various molecules such as sHLA-G5, IL-10, IL-35 and transforming growth factor (TGF) -β involved in the immune tolerance process. In addition, some normal resting and activated CD4+ and CD8+ T cells may acquire HLA-G1 from APCs as a result of trogocytosis (the unidirectional and active process of transferring membranes and related molecules from one cell to another molecule between contacting cells), which allows them to change their functions from effector cells to regulatory ones. In addition, this process has also been described for monocytes and NK cells [61]. HLA-G1 trogocytosis represents a novel mechanism for the production of regulatory cells: one that does not involve the maturation of long-lived regulator cells but involves the direct and immediate conversion of effector cells into cells that acquire an effective but temporary regulatory function in situ and thus provides local emergency immune suppression [62,63]. Among the most abundant immune cells found in the mother’s endometrium, their initial amount varies between 20 and 30% at the place of implementation, gradually increasing their level throughout the entire pregnancy. Studies have shown that during ligation with HLA-G molecules, the functions of monocytes and macrophages may also be altered. During a properly developing pregnancy, IFNγ, released by maternal NK cells, activates monocytes and macrophages. These molecules then interact with trophoblast cells and bind HLA-G to ILT2 and ILT4 receptors, which leads to decreasing the ability of monocytes and macrophages to lyse cells and induce apoptosis. This process is also modulated by cytokines produced by Th1 and Th2. Afterwards, the production of cytokines by Th2 increases and the number of cytokines produced by Th1 decreases. The activity of monocytes and macrophages may not be silenced during pregnancy, and a compilation occurs. It concerns the inability to bind the HLA-G molecule with ILT2 and ILT4 receptors on maternal immune cells, as it is a result of the lack of expression of HLA-G molecules on the trophoblast. At this point, the activity of monocytes and macrophages is not suppressed, and the trophoblast becomes impaired. These changes also influence the production of cytokines by Th1 and Th2. In this case, the production of cytokines by Th1 cells is increased and by Th2 cells is decreased [64,65,66].

**Table 3:** Selected examples of the influence of the HLA-G immunomodulating molecule on immune cells.

Name of Immune Cell	Tolerogenic Function of HLA-G	Reference
T cells	Inhibition of proliferation, cytolisys, and chemotaxis, cytotoxicity, and INF-y secretion	[67,68,69]
	Induction of Treg, cytokine produced by Th2	[70]
B cells	Inhibition of proliferation, Ig secretion, and chemotaxis	[71,72]
Neutrophils	Inhibition of phagocytosis and reactive oxygen species production	[73]
Dendritic cells	Inhibition of maturation and NK cell activation	[54,74]
	Induction of anergic and suppressor T cells, and tolerogenic DC	[54,75,76]
NK cells	Inhibition of chemotaxis, cytotoxicity, and INF-y secretion	[77,78]

## 4. Selected Autoimmune Diseases Which Involve the HLA-G Molecule

The activity of the human immune system is extremely complex and is regulated by several environmental or genetic factors. It provides protection against infection and pathogen-induced inflammation. The immune system has also developed a tolerance to its own antigens, while its disturbances may lead to the deregulation of its functioning, following the development of auto-aggression, which is the basis of autoimmune diseases [79]. Literature data from numerous reports indicate that, every year, the number of people suffering from this type of diseases is increasing. It is estimated that, by 2025, the number of diagnosed autoimmune disorders in Europe will rise by nearly 7.8% compared to 2020 [80]. As indicated by European studies on the number of autoimmune diseases, the incidence of multiple sclerosis has increased from 83,000 to 100,000, nearly 2 million people suffer from rheumatoid arthritis, and approximately 2–3% of the entire population suffer from psoriasis, which is one of the most common forms of autoimmune diseases [80]. Despite the growing awareness among the doctors and the public about these diseases, their etiopathogenesis is still not fully understood, and scientific research shows us only a small part of the processes taking place in the patient’s organism. This is why it is so important to search for new markers, molecules, and mechanisms that result in the development of autoimmunity. As indicated in the literature, one of such molecules may be HLA-G, which, due to its immunomodulatory properties and the possibility of interaction with other immune cells, may affect the immune system functioning [10].

### 4.1. The Role of the HLA-G Molecule in the Pathogenesis of Autoimmune Diseases of the Gastrointestinal Tract

#### 4.1.1. Influence of the HLA-G Molecule on the Development of Celiac Disease

One of the most common autoimmune diseases of the digestive system is enteropathy with gluten sensitivity, known as celiac disease. It is caused by an altered immune response in contact with gluten and its associated prolamines, which occurs in people with a genetic predisposition, understood as the presence of HLA-DQ2 and/or HLA-DQ8 histocompatibility antigens [81,82,83]. Research shows that 95% of patients with celiac disease have the HLA-DQ2 haplotype, and the remaining 5% have the HLA-DQ8 haplotype. The positive result is considered to be the presence of two alleles corresponding to the coding of two chains: alpha (DQA allele) and beta (DQB allele). In the case of having only one allele, such a person is considered as a carrier, which, according to scientists, may contribute to predisposition to celiac disease. In addition, the risk of this disease in close relatives ranges from 5 to 15%, while in the ones with genetic predisposition, the risk increases and ranges from 10 to 30% [67]. At the root of the development of celiac disease is the immune system’s response to dietary gluten. From a molecular point of view, not all gluten can be immunized, but only a 33 amino acid fragment (located in the N region of alpha-gliadin between amino acids 57 and 89). It is the most toxic and immunizing fragment because of its resistance to the digestive enzymes located in the stomach. As a result, it can be effectively captured, presented and bound by cells of the HLA-DQ2 and/or HLA-DQ8 system [68]. Data from genetic analyses of patients with celiac disease showed that there are characteristic polymorphisms of the HLA-G gene, which indicate an increased susceptibility to the disease in these people. Five polymorphisms were found: −477 C > G, −369 C > A, 14 bp del/ins, 3187 A > G, 3196 C > G and one haplotype (TCGGTACGAAITCCCGAG), which occurred significantly more often in patients with celiac disease than in healthy subjects [69].

#### 4.1.2. The Role of HLA-G in the Development of Crohn’s Disease

Crohn’s disease is an autoimmune disease in which recurrent inflammation occurs along almost entire gastrointestinal tract. It may intermittently extend to any part of the gastrointestinal tract, especially the terminal ileum and/or the rectum. Statistical data estimate that as many as 500,000 people in North America could suffer from this disease, while in Asian countries it may affect up to 7.7 per 100,000 people [70,71]. Scientific research suggests that the HLA-G molecule may also be involved in its pathogenesis. Several hypotheses have been suggested that could explain how the variability of HLA genes influences the development of this disease. The first one concerns the role of HLA-G in presenting endogenous antigens (derived from viruses or bacteria present in the intestine) that trigger the development of the autoimmunity through molecular mimicry. Then, auto-reactive T lymphocytes are activated, which, acting as superantigens, stimulate a large number of T lymphocytes [72]. The second hypothesis concerns changes in HLA class I and II expression levels, which potentially lead to greater antigen presentation to CD8+ and CD4+ T cells, in correlation with some more disease-prone alleles [73]. The third hypothesis relates to the role of HLA class I molecules in the process of inhibiting the activity of NK cells by controlling the balance between activating and inhibiting receptors on their surface [74]. HLA typing plays an important role in the diagnosis of Crohn’s disease. The researchers suggest that epigenetic changes in the DNA of patients with this condition may provide the necessary information about disease development in the context of the interaction between genetics and environmental factors, such as diet and gut bacteria. HLA-G expression was demonstrated on the surface of the mucosa cells of ulcerative colitis (UC) patients, while it was not found in patients with Crohn’s disease and the control group [75]. In studies conducted by Rizzo et al., peripheral blood mononuclear cells (PBMCs) from patients with Crohn’s disease have been discovered to spontaneously secrete sHLA-G, whereas cells from UC patients and controls do not. Moreover, the stimulation of cells with bacterial lipopolysaccharide from Crohn’s disease patients led to the increased production of sHLA-G and IL-10, compared to subjects with UC. Based on the conducted research, the authors postulate that the insufficient secretion of IL-10 and HLA-G may lead to the development of inflammatory changes in ulcerative colitis [76].

### 4.2. The Role of HLA-G in the Development of Multiple Sclerosis

Another analyzed autoimmune disease is a chronic demyelinating disease of the central nervous system, which is multiple sclerosis (MS). In its development, characteristic damage to the nervous tissue is observed, particularly changes in the form of demyelination and axonal breakdown. Although MS is not considered a hereditary disease, an increasing number of studies indicate that genetic factors are significantly involved in its pathogenesis, determining personal predisposition to the disease. Analyses of genealogies of people suffering from MS have shown that in the families of patients diagnosed with more than one incident, certain regions of genes become more often inherited than in the general population. Numerous genetic studies based on the analysis of the genomes of patients with MS have shown that up to 200 loci may be involved in the pathogenesis of this disease, of which scientists attribute the most important role to the genes of the main HLA histocompatibility complex on chromosomes [77,78,84]. As indicated by the researchers, one of the most important genetic determinants of the development of MS, determining both the predisposition or resistance to the disease, is the HLA class II gene DRB1 (haplotype HLA-DRB5*0101-HLA-DRB1*1501-HLA-DQA1*0102 -HLA-DQB1)*0602). Due to the high polymorphism of this gene among patients with MS, scientists observe the occurrence of genetic heterogeneity, which correlates with the geographical variability of the population. For example, Japan is dominated with the HLA-DRB1*09 allele, Spain with HLA-DRB1*03 and HLA-DRB1*14 alleles, and Canada with HLA-DRB1*01, HLA-DRB1*03, HLA-DRB1*08, HLA-DRB1*10, HLA-DRB1*11 or HLA-DRB1*14 alleles [84]. Despite the discovery of HLA peptides as ligand molecules (MBP85-99, MBP86-105, MBP111-129 peptide) recognized by T lymphocytes, the full mechanism of HLA’s contribution to MS is not fully understood. HLA class I genes are also involved in the development of MS. Studies have shown that the occurrences of HLA-A*02, HLA-B*12-HLA-Cw*05, and HLA-A*02-HLA-B*44-HLA-Cw*05 are associated with a reduced risk of developing MS [85,86,87].

The initial form of sHLA, which has been detected in the cerebrospinal fluid of patients suffering from relapsing–remitting MS, also plays an important role. It has also been shown that higher levels of sHLA-G in the cerebrospinal fluid are associated with IL-10 values in these patients, which may suggest that levels of sHLA-G in the cerebrospinal fluid are responsible for modulating disease activity, acting as anti-inflammatory molecules along with IL-10 [88].

Research data indicated that HLA-G molecules in patients with MS treated with INF-β are also responsible for inhibiting the proliferation of CD4+ T cells or the production of pro-inflammatory cytokines by Th1 and Th2 lymphocytes. In addition, researchers suggested that HLA-G levels in postpartum serum are higher in patients with asymptomatic MS, what could be related to the possible immunomodulatory effect of HLA-G on MS activity in pregnancy [65,89]. According to Wiendl H. et al. in their studies of microglia, macrophages and endothelial cells located in MS lesions showed a significant expression of HLA-G and its inhibitory receptors for ILT-2 and ILT-4. On the other hand, the activation of Th1 pro-inflammatory cytokines in the culture of human microglia cells in MS patients confirmed the increase in HLA-G expression [89]. In addition, researchers characterized a new subpopulation of naturally occurring CD4+ and CD8+ T cells expressing HLA-G (HLA-Gpos Treg) in the peripheral blood of MS relapsing patients, showing inhibitory effects through HLA-G5 secretion and sHLA release-G1 [60,90]. In conclusion, the collected data confirmed the association with the immunosuppressive activity of HLA-G antigens (particularly the HLA-G-5 isoform), HLA-G-positive Treg lymphocytes and autoimmunity remission in MS [91]. Additionally, the presence of HLA-G gene polymorphisms in patients with multiple sclerosis significantly correlates with the incidence of the disease. Fredj et al. showed that the genotype of the 14 bp DEL/+3142 G haplotype is more common in patients with MS than in the control group [92]. Similar conclusions were reached by Wisniewski et al., who showed that the presence of polymorphisms: −725C > G > T and −716T > G, as well as the 14 bp INS/DEL HLA-G was associated with an increased susceptibility to this disease in the Polish population [93]. These studies suggest that the risk of MS is associated with only a single allele but may also depend on the presence of HLA polymorphisms. However, studies up to date do not explain the relationship between sHLA-G and the progression of MS (disability progression, MRI changes). The collected data encourage further research for prognostic and diagnostic markers that monitor disease activity and response to therapy in MS patients.

### 4.3. The Role of HLA-G in the Development of Rheumatoid Diseases

#### 4.3.1. The Role of HLA-G in the Development of Rheumatoid Arthritis

Rheumatoid arthritis (RA) is a systemic autoimmune disease of connective tissue characterized by symmetrical nonspecific arthritis affecting mainly small and medium-sized joints and accompanied by systemic complications. The development of the disease leads to joint destruction, deformities, contractures, and impaired motor functions, which ultimately causes progressive disability and premature death. Treatments for RA include disease-modifying drugs (DMARDs) and biological agents [94]. Literature data suggest the involvement of immune mechanisms mediated by HLA-G in the development of the disease. One of the mechanisms leading to the onset of symptoms in RA patients is an autoimmune reaction in the synovial cells of the joints. In one study, the levels of sHLA-G molecules have been checked at the site of synovitis and higher levels of sHLA-G have been found in RA patients. The increase in HLA-G may be related to the recruitment of activated HLA-G+ immune cells and the local production of activated synovial fibroblasts [95,96]. In addition, RA patients had significantly lower serum levels of sHLA-G than the control group, which may affect the chronic activation of inflammatory cells and the development of chronic inflammation [97,98]. Surprisingly, Catamo et al. showed that sHLA-G levels in the synovial fluid of juvenile idiopathic arthritis patients were found to be higher than in the control group [99]. The HLA-G gene polymorphism may also play an important role in the development of the disease. The results published by Hashemi et al. showed the association between HLA-G+ 3142G > C polymorphism and RA occurrence in patients from the Iranian population [100]. This study was also confirmed by Gautam et al. [101]. Literature data of the meta-analysis by Lee et al. did not show a significant relationship between 14 bp HLA-G I/D and the +3142G/C polymorphism and the risk of developing RA [102]. Similar negative results were recorded in the Brazilian and Indian populations [99,103]. The 14 bp HLA-G INS/DEL polymorphism was assessed as a pharmacogenetic marker of therapy with Methotrexate (MTX), which is one of the anti-rheumatoid drugs that allows to modify the course of the disease. Research data show that its usage results in the increased production of IL-10 and increases the secretion of HLA-G by peripheral blood mononuclear cells [104,105]. After 6 months of treatment with MTX, the incidence of the 14 bp HLA-G INS/DEL polymorphism was increased among patients. However, not all studies available in the literature confirm this tendency, which may be related to the use of different doses of the drug in the studies [106,107,108].

#### 4.3.2. The Role of HLA-G in the Development of Systemic Scleroderma

Another autoimmune rheumatic disease is systemic scleroderma (SSc). In the course of this disease, the structure and function of blood vessels are disturbed, accompanied by the fibrosis of the skin and internal organs, gradually leading to their failure; however, the etiology of SSc is not yet known. The literature draws attention to the coexistence of genetic (HLA DQ7, DR2), hormonal, immunological and environmental factors, such as exposure to chemicals or infectious agents [109]. Wastowski et al. demonstrated for the first time HLA-G expression in lesion sites (epidermal cells and dermis) in skin biopsies from SSc patients. As their experiments show, HLA-G molecules were observed in the keratinocytes of the basal and suprabasal layers of cells, which are involved in many immune and homeostatic mechanisms of the skin. In the dermis, HLA-G molecules were observed in eccrine sweat glands, which are completely trapped by broad collagen fibers during SSc progression [109]. Additionally, the researchers’ correlation of clinical features with the presence of HLA-G showed that HLA-G expression was associated with a lower incidence of skin lesions (calcinosis and telangiectasia), changes in blood vessels (ischemic skin ulcers) and changes in internal organs [109,110]. Studies conducted by Negrini et al. showed that the percentage of HLA-G-positive monocytes is increased in both SSc and SLE patients. Moreover, they also showed that the percentage of CD4+ and CD8+ cells expressing HLA-G molecules was significantly higher in SSc and SLE patients than in controls [110]. The studies presented in the literature show that HLA-G molecules are involved in the immune response at the skin level.

#### 4.3.3. The Role of HLA-G in the Development of Systemic Lupus Erythematosus

Another autoimmune disease involving HLA-G molecules is systemic lupus erythematosus (SLE). The development of this disease occurs as a result of an abnormal response of the immune system resulting from the loss of tolerance to its own antigens, the production of autoantibodies, and the formation and deposition of immune complexes in tissues. As a result, the cytokine system is activated, leading to tissue destruction and the development of end-stage multi-organ failure [111]. Studies conducted by Rosado et al. and by Chen et al. have shown that SLE patients have higher levels of sHLA-G compared to healthy subjects, while Rizzo et al. observed lower levels of sHLA-G in SLE patients [37,112,113]. The different results of these studies may be due to the difference in the analyzed samples’ serum or plasma, since the highest levels of sHLA-G were found in the plasma samples compared to the serum collected from the same patients. This is due to a trapping phenomenon during clot formation, which can subtract sHLA-G from the serum [114]. Monsiváis-Urenda et al. analyzed the monocytes and CD83+ dendritic cells of patients diagnosed with SLE—the study showed decreased HLA-G expression compared to healthy controls. Moreover, the analysis of monocytes from SLE patients showed decreased HLA-G expression in response to IL-10, and lymphocytes showed lower HLA-G acquisition from autologous monocytes compared to the controls [115]. The increased expression of the ILT-2 receptor was also demonstrated on CD3+, CD19+, CD56+ lymphocytes, and on anti-DNA antibodies bound to IL-10. The advantageous use of the SNP mapping approach contributed to the definition of a new independent site for SLE [113]. Literature data also suggest the involvement of the 14 bp INS/DEL HLA-G and SNP HLA-G +3142 C > G polymorphisms in the development of SLE [116]. The incidence of the 14 bp INS allele and the 14 bp INS/INS genotype was shown to be higher, and the association was demonstrated with the presence of 14 bp INS/INS and increased disease activity [117,118]. On the other hand, studies assessing the HLA-G 14 bp INS/DEL polymorphism in the Brazilian SLE population did not show any relationship, while the +3142G allele and the +3142 GG genotype frequency were increased in SLE patients compared to the control group [118,119,120]. These differences were explained by the heterogeneous picture of clinical patients qualified for the study, as well as the effect of the pharmacotherapy used. The collected data confirm the important role of HLA-G molecules in modifying the SLE state, and in addition, some of them confirm the lower expression of HLA-G as a factor in the development of SLE.

#### 4.3.4. The Role of HLA-G in the Development of Behçet’s Disease

Behçet’s disease (BD) belongs to a group of diseases called systemic vasculitis. Literature data indicate that the etiology and pathogenesis of this disease entity has not yet been clearly defined. However, it is believed to be caused by genetic, environmental, and autoimmune factors [121]. The presence of HLA class I and class II alleles is considered to be important, including HLA-A26, HLA-B15, HLA-B5701, HLA-B2702, HLA-B3901, HLA-B52, HLA-B56, Cw1, Cw14, Cw15, Cw16, HLA-DRB104, and HLA-DRB107 in the prevalence of BD in various populations. Some scientists have shown that there are links between HLA-E, HLA-F, and HLA-G gene polymorphisms and the incidence of BD in Korean and Japanese patients. However, the studies performed included not only selected populations, but also a small number of patients; therefore, the presented significance was small and occurred in individual studies [122,123,124,125]. A study by Sakly et al. revealed that the HLA-G 14 bp ins/del polymorphism appears to be related to BD, as the incidence of the 14 bp allele is higher in patients compared to healthy controls. In addition, higher plasma levels of sHLA-G during active BD have also been found [126]. The researchers concluded that the different alleles of HLA-G genes have different effects on the risk of developing BD—the HLA-G* allele 01:01:01 is associated with a reduced risk of developing BD, while HLA-G* alleles 01:01:02 and G*01:05N are associated with an increased risk of developing BD [127,128].

#### 4.3.5. The Role of HLA-G in the Development of Kawasaki Disease

Another discussed autoimmune disease is Kawasaki disease. It is an acute inflammatory disorder of unknown etiology associated with the inflammation of the blood vessels, aneurysm formation, and myocardial ischemia, making it the main cause of acquired heart disease among children in developed countries [129]. It is estimated that the pathogenic nature of this disease involves many environmental factors, the development of infection, as well as the individual predisposition, including age and genetic factors [130]. According to the literature, the prevalence of this disease in the population is quite low, therefore, all studies conducted on patients suffering from these diseases have a small sample size. Jae-Jung Kim et al. showed a relationship between the non-synonymous SNP (+755A/C) of the HLA-G gene (rs12722477, G*01:04) with the occurrence of Kawasaki disease [10]. The risk of this disease is believed to be related to the long-term regulation of HLA-G expression. However, the mechanism of regulation is still unknown, so scientists call for increased research to determine the presence of HLA-G polymorphisms in populations other than the one studied by Jae-Jung Kim et al. Perhaps this will allow the use of the HLA-G polymorphism as a diagnostic marker for Kawasaki disease in the future.

### 4.4. The Role of HLA-G in the Development of Selected Respiratory Diseases

#### 4.4.1. The Role of HLA-G in the Development of Asthma

Asthma is a chronic heterogeneous inflammatory disease of the airways characterized by multiple and recurrent symptoms, such as coughing, dyspnea, reversible airway obstruction, and bronchospasm. Statistics from 2019 estimate that it affects approximately 262 million people worldwide and causes approximately 400,000 deaths each year. The greatest number of cases (within 20% of the population) was recorded in developed countries, including Great Britain, Australia, Sweden, Finland, and the lowest (within 1% of the population) among Inuit and Japanese [131]. Nicolae et al. suggested the role of the HLA-G gene as an asthma susceptibility gene [132]. The research stresses that HLA-G expression is not present in all somatic cells but is limited to selected sites in the human body, including human bronchial epithelial cells (HBECs). The presence of HLA-G5 has been demonstrated in the epithelium of the respiratory tract and in the bronchoalveolar lavage fluid of patients with asthma [133,134]. The development of asthma is characterized by abnormalities in the bronchial epithelium and the activation of inflammatory cells, which, as research has shown, is also associated with the HLA-G genetic polymorphism [135]. This is also confirmed by the studies conducted by Tan et al., exploring the relationship between +3142 C > G (rs1063320) and the incidence of asthma [136]. As indicated in the literature, the HLA-G molecule also affects the type of asthma occurring. The presence of higher plasma levels of sHLA-G has been demonstrated in children with atopic asthma compared to children without atopic symptoms and without asthma [134]. However, the 14 bp INS/DEL polymorphism has not been shown to influence the plasma levels of sHLA-G in children with atopic asthma. In vitro studies have indicated that the presence of HLA-G may differ from the physiological status in asthma. Patients with isocyanate-induced asthma have increased the expression of sHLA-G compared to patients with classical asthma [137,138]. The research indicates that the HLA-G molecule may be an important biomarker of asthma development and potentially more broadly influencing the immune system, especially in the context of modulating local immunity in the mucosa.

#### 4.4.2. The Role of HLA-G in the Development of Allergic Rhinitis

The underlying cause of allergic rhinitis (AR) are immunoregulatory disorders with reduced tolerance to allergens [139,140]. From an immunological point of view, the development of AR is associated with the development of IgE-mediated inflammation, which is maintained by Th2 cells. Mucositis is also characterized by the activation of mast cells and eosinophilia [141,142]. Due to their immunosuppressive properties, sHLA-G molecules may play also an important role in the mechanisms of immune tolerance to allergens during AR. Studies conducted both in the pollen season, outside the pollen season, and in patients with perennial allergic rhinitis clearly showed an increased level of sHLA-G molecules in sera [143,144,145]. In their research, Ciprandi et al. found that sublingual immunotherapy (SLIT) in allergic rhinitis is able to lower serum sHLA-G levels in pollen-allergic patients, which may suggest clinical implications as a biomarker of SLIT response [146,147]. Another interesting observation is the significantly higher level of sHLA-G molecules in children with allergic rhinitis than in healthy controls or in children with allergic asthma [148].

### 4.5. The Role of HLA-G in the Development of Diabetes

Type 1 diabetes mellitus (T1DM) is a chronic autoimmune disease resulting from the destruction of β cells, usually leading to complete insulin deficiency. One of the genetic risk factors that studies have shown is the HLA gene [149]. Cirulli et al. showed that HLA-G is constitutively expressed in the endocrine compartment of the human pancreas, particularly in the secretory granules of insulin. Moreover, it may be upregulated on the surface of primary pancreatic islet cells that have been stimulated to secrete insulin. HLA-G may prevent the activation of autoreactive T cells, potentially by regulating immunogenic ligands present at insulin exocytosis sites [150]. The INS/DEL 14 bp polymorphism in HLA-G 3ʹUTR may influence diabetes’ susceptibility, thus suggesting a new candidate gene. The upregulation of sHLA-G isoform has been detected in pre-diabetic and diabetic patients, suggesting a role in metabolic dysfunction [151,152]. Research conducted by Silva et al. and Gerasimou et al. demonstrated the implications of 14bp INS/DEL HLA-G polymorphisms for T1DM susceptibility and concluded that the homozygous deletion genotype was associated with an earlier age of onset. The presence of the DEL/DEL genotype in the early onset of T1DM was increased almost three times compared to the late onset patients. These data strongly suggest that the DEL/DEL genotype is strongly associated with the earlier expression of autoimmune diabetes [153,154].

Solini et al. conducted research aimed at determining the role of HLA-G molecules in the development of obesity, one of the consequences of which is the presence of type 2 diabetes [155]. Their research was based on the analysis of the relationship between the HLA-G molecule and the metabolic and inflammatory pattern found in obesity and diabetes, and the varying degrees of glucose tolerance in these patients. They showed that there is a relationship between the degree of glucose tolerance and increased levels of sHLA-G in the plasma of patients (considering differences due to age, gender or correlation with metabolic parameters). Additionally, researchers found that HLA-G is directly related to IL-6, which is involved in the inflammation associated with the course of obesity and type 2 diabetes. Scientists argue that although type 2 diabetes is not an autoimmune disease, increased levels of sHLA-G in the plasma of patients determine the development of metabolic abnormalities in these patients. This is also confirmed by the studies carried out by Solini et al. showing the correlation between blood pressure, cholesterol levels, and HLA-G. Despite the lack of evidence of impaired innate humoral immunity in type 2 diabetes, the researchers showed significant changes in the immune system functioning that correlated with higher levels of sHLA-G in the plasma of patients. These changes concerned the functional impairment of diabetic multinucleated cells as well as monocytes and macrophages compared to controls [155]. The involvement of HLA-G molecules in glucose tolerance has also been confirmed by other scientists, including Saeide-sadat Shobeiri et al. [156], Abdel Hameed et al. [157], and Oztekin et al. [158] in the course of gestational diabetes.

### 4.6. The Role of HLA-G in the Development of Psoriasis

Psoriasis is a complex skin disease in which the skin lesions are characterized by sharply delimited, highly scaly plaques. These changes are the result of epidermal hyperplasia induced by T cells, capable of producing interleukins (IL)-17A, IL-22 and IFN-γ. The literature data show that the disease is caused by a wide variety of environmental, genetic, infectious, and even lifestyle factors [159,160,161]. Additionally, there is an evidence in the literature that HLA molecules are involved in the progression of psoriasis. The studies conducted by Nair et al. [162] and Zhou et al. [163] suggest that the HLA-C*06:02 allele may be one of the major genetic factors associated with the incidence of psoriasis patients. These observations are also confirmed by the results of the Cassia syndrome study, in which, in addition to the role of the HLA-C*06:02 allele, the participation of other alleles such as HLA-B*57 (significantly increased in patients, indicating the risk of developing psoriasis) and HLA-C*8 (high values found only in patients with psoriatic arthritis) was shown [164]. Very little information in the literature was found regarding the role of the HLA-G molecule in the development of this type of disease. The studies conducted by Borghi et al. showed significantly lower levels of both sHLA-G and IL-10 in the plasma of patients diagnosed with psoriasis than in the control group. According to the authors, this may indicate the susceptibility of the respondents to psoriasis. However, genetic analysis by scientists showed no significant differences in the frequency of HLA-G alleles between patients and controls [165]. Due to the scarcity of studies available in the literature on the role of HLA molecules in the pathogenesis of psoriasis, it seems extremely important to undertake new comprehensive analyses that could explain their impact in the course of this disease.

### 4.7. Role of HLA-G in Uveitis

Ocular inflammation is a disease that is quite rare in the population (115–204 cases per 100,000 population) but poses a serious threat to vision loss. As reported in the literature, one of the etiopathological factors of the development of this disease are infections that can directly or indirectly (by activating innate immune processes against infection) cause tissue damage [166,167,168]. This type of disease is recognized by specialists as an infectious type of eye inflammation. The second type of the disease is the non-infectious form, which is not synonymous with autoimmune uveitis. There are conflicting opinions among many scientists regarding the autoimmune basis of this type of eye inflammation. One of the hypotheses explaining the role of the immune response (more specifically the autoimmune response) comes from experimental studies conducted on models of retinitis that break down the blood–retinal barrier (BRB) and stimulate acquired immunity directed at antigenic targets of the retina [166]. The pathogenesis of uveitis is not fully understood. Moreover, some of the scientific research available in the literature indicates the involvement of the immune system in the development of this disease. It is known that eye trauma can cause cell damage (or death), with the consequent release of inflammatory cytokines and the development of inflammation, which is mediated by CD4 Th1 cells. Usually, only activated lymphocytes pass the blood–retinal barrier reducing the sensitization of naive T cells to the eye proteins [169]. As research on this topic developed, some scientists suggested that HLA molecules may be involved in the pathogenesis, which is possible due to the formation of molecular mimicry between retinal S–Ag peptides and HLA-B, leading to the development of an inflammatory response [169,170]. This was confirmed in the studies of Huang et al. and Sanjanwal et al., where it was shown that the amino acid positions located in the site responsible for binding the HLA-B antigen (precisely positions 116, 67, and 97) are important determinants that affect the affinity of the HLA-B alleles for KIR3DL1 receptors (killer cell immunoglobulin like receptor, three Ig domains and long cytoplasmic tail 1) and KIR3DS1 (killer cell immunoglobulin like receptor, three Ig domains and short cytoplasmic tail 1). These findings further imply the binding of peptides to MHC molecules in the pathogenesis of non-infectious uveitis [171,172]. The importance of the HLA-G molecule in the pathogenesis of ocular inflammation is not fully understood. From the few literature reports and studies conducted by Crabtree et al., it appears that the HLA-G molecule may be involved in the pathogenesis of non-infectious uveitis in rats. In their research, scientists proposed a new approach to the long-term therapy of this disease with the use of gene therapy based on adenovirus (AAV), the presence of which will allow the use of the natural mechanism of HLA-G-induced immune tolerance (HLA-G-1 and HLA-G-5). The induction of immune tolerance by the HLA-G molecule takes place directly by inhibiting the interaction of HLA-G1 with almost all cells of the immune system and indirectly through the soluble HLA-G5 isoform, the combination of which is to prevent the recognition of its own antigen. Studies have shown the AAV-dependent expression of HLA-G-1 and -5 transgenes in ocular tissues after a single intravitreal injection of AAV-HLA-G1/5 significantly reduced the resulting inflammation in rats. Therefore, as researchers suggest, the local delivery of AAV-HLA-G1/5 genes to the eye may reduce the risk of developing inflammation and establish a long-term immunosuppressive effect that would serve as an effective and new therapeutic strategy [173].

## 5. Conclusions

The data collected and presented in this review article were intended to emphasize the significant influence of HLA-G molecules on the development and course of selected autoimmune diseases, including the gastrointestinal tract, respiratory system, rheumatoid diseases, and type 1 diabetes mellitus. The collected data indicate that HLA-G is an important natural tolerance-inducing molecule that is being widely studied in a pathological context in adults and children. However, despite the increasing amount of research on the role of HLA-G, new aspects, mechanisms and interactions are still being discovered, indicating the unusual complexity of the functioning of this molecule in the human body. In fact, HLA-G is no longer considered a monomorphic, classical HLA class I-like molecule that acts as a shield against immune aggression. The numerous structural changes, the presence of polymorphisms and the complex network of regulation of the expression of this molecule indicate that its function goes far beyond the long-term and local mechanisms of immune tolerance.

## Figures and Tables

**Table 2:** Receptor characteristics for HLA-G antigens considering the expression on selected cells of the immune system and their interactions based on [2,39,40].

Receptor	Gene	Protein Mass (kDa)	The Number of Amino Acids	Expression							Interactions
				NK	CD4+ T Cell	CD8+ T Cell	B Cell	Monocytes	Macrophage	DC	
ILT2	*LILRB1*	65,039	598	some	some	some	+	+	+	+	α3+β2m (dimer)
ILT4	*LILRB2*	44,601	400	-	-	-	-	+	+	+	α3 (dimer)
KIR2DL4	*KIR2DL4*	41,487	377	+	some	-	-	-	-	-	α1
CD8	*CD8B*	21,524	192	some	+	-	-	-	-	-	α3
CD160	*CD160*	19,810	181	+	+	+	-	-	-	-	-
